# Through thick and thin: how optical cavities control spin

**DOI:** 10.1515/nanoph-2023-0175

**Published:** 2023-05-05

**Authors:** Jefferson Dixon, Feng Pan, Parivash Moradifar, Priyanuj Bordoloi, Sahil Dagli, Jennifer Dionne

**Affiliations:** Mechanical Engineering, Stanford University, 440 Escondido Mall, 94305, Stanford, CA, USA; Materials Science and Engineering, Stanford University, 496 Lomita Mall, 94305, Stanford, CA, USA

**Keywords:** chirality, high-Q, metasurfaces, photonic crystals, spin

## Abstract

When light interacts with matter by means of scattering and absorption, we observe the resulting color. Light also probes the symmetry of matter and the result is encoded in its polarization. In the special case of circularly-polarized light, which is especially relevant in nonlinear optics, quantum photonics, and physical chemistry, a critical dimension of symmetry is along the longitudinal direction. We examine recent advances in controlling circularly-polarized light and reveal that the commonality in these advances is in judicious control of longitudinal symmetry. In particular, in the use of high quality-factor modes in dielectric metasurfaces, the finite thickness can be used to tune the modal profile. These symmetry considerations can be applied in multiplexed optical communication schemes, deterministic control of quantum emitters, and sensitive detection of the asymmetry of small molecules.

## 1 Introduction

Optical cavities trap light, akin to a mirrored box. As the light bounces around the box, the probability that the photons interact with other particles inside the box is increased. The increased interaction time allows otherwise weak light–matter interactions to become appreciable, which amplifies weak nonlinear optical processes (e.g., laser cavities) and may aid in developing photon sources for quantum calculations (“qubits”). Critical to controlling the light–matter interactions in an optical cavity is determining both the wavelength (dictated by the size of the cavity) and polarization (dictated by the symmetry of the cavity) of trapped light.

Symmetry in two dimensions (i.e., a box with no lid) is sufficient for controlling states of linearly-polarized light, but the third dimension (the lid) plays a unique role in controlling states of circularly-polarized light. We see this at the macroscale with bulk polarization optics: a wire grid polarizer is sufficient to filter linearly-polarized light, but a wave plate with finite thickness is required to filter circularly-polarized light. This is because circularly-polarized light passing through a filter (in the *z*-direction) possesses both angular momentum (in *x*–*y*) and linear momentum (along *z*). In optical cavities, light is not always propagating and yet there is still angular momentum [1]. In these cases, it is important to distinguish between the spin of light and the chirality of light. Chirality is the temporal rotation (spin angular momentum in *x*–*y*, or simply spin) projected along the propagation direction (linear momentum in *z*); therefore, spin can be defined in two dimensions while chirality requires all three dimensions [2–4].

**Figure 1:**
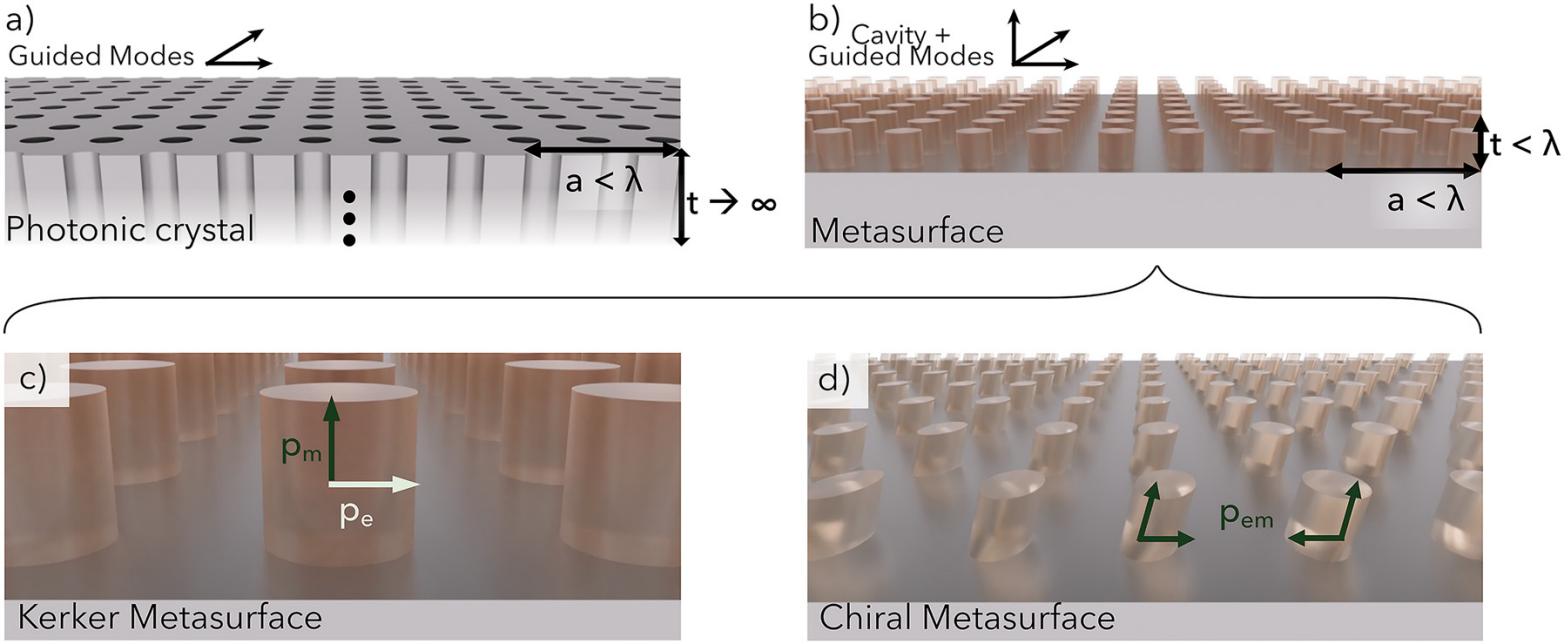
Dimensions in the longitudinal (*t*) and transverse (*a*) directions for (a) a photonic crystal and (b) a dielectric metasurface. Here, a is the lattice period, *t* is the thickness, and *λ* is the effective wavelength of light. Electromagnetic dipole moments in (c) a Kerker metasurface with orthogonal but overlapping electric and magnetic dipole moments (*p*_e_ and *p*_m_), and (d) a chiral metasurface with coupled electric and magnetic dipole moments (*p*_em_).

The promise of photonic crystals as they were originally described in 1987 was to confine light in all three spatial dimensions, creating a complete bandgap (i.e., a span of energies over which light is completely trapped) regardless of polarization [5]. In practice, such photonic crystals have been traditionally made of bulk crystals that are effectively semi-infinite in their thickness, with periodically etched holes introduced in the transverse direction (Figure 1a), resulting in two-dimensional confinement [6]. Thereafter, the development of plasmonic (i.e., metallic) arrays reduced confinement in the longitudinal direction towards zero (because of the finite “skin depth” penetration of light into metals) [7–11]. The emergence of dielectric metasurfaces introduces a finite degree of confinement in the longitudinal direction while retaining the periodic confinement in the transverse direction (Figure 1b), approaching quasi-three-dimensional confinement [12].

Metasurfaces shape the amplitude and phase of light in the transverse direction, but the longitudinal direction can still be exploited to control states of circularly polarized light (CPL). Such control of CPL has important implications for nonlinear optics, quantum photonics, and beyond [13, 14]. For example, in the case of quantum emitters, these metasurfaces can be used to control the amplitude, phase, and spin of their emission while simultaneously optimizing their coherence. Metasurfaces can be interfaced with a variety of media, including van der Waals two-dimensional materials (e.g., 2D transition metal dichalcogenides) that exhibit a valley degree of freedom for valleytronics, lattice point defects with spin inherited from trapped electrons for color centers, and chiral molecules with symmetry-selective absorption for enantioselective photo-destruction [15–17]. Dielectric metasurfaces possess lower loss than their metallic counterparts and can achieve stronger temporal confinement with high quality-factors (high-Qs) for stronger interactions. In this perspective, we explore the longitudinal degree of freedom in optical cavities, its role in controlling circularly-polarized light in high-Q metasurfaces, and discuss opportunities in interfacing with quantum emitters.

## 2 Metasurface thickness allows magnetic-type Mie modes

The number of communication channels for a given optical element is equivalent to the number of orthogonal modes that can be solved for in that element [18]. For photonic crystals with semi-infinite thickness, these modes rely on translational symmetry in the periodic direction (i.e., guided modes). For dielectric metasurfaces with finite thickness, there also exist modes without translational symmetry (i.e., cavity modes) in addition to the usual guided modes [19, 20]. Metasurface cavity modes exist for individual dielectric particles and are described using Mie theory, while the guided modes result from the periodic potential and are described using Bloch's theorem. In terms of their energy-momentum relationship, guided mode dispersion is approximately linear while cavity mode dispersion is approximately flat, and cavity dispersion is trivial for a geometry lacking translational symmetry on the order of the wavelength (i.e., there is no reciprocal lattice for a single particle). The dispersion of high-contrast gratings such as metasurfaces convolute the local (cavity) modes into a nonlocal (guided) envelope, resulting in parabolic dispersion that is flat near high-symmetry points and linear away from high-symmetry points [21]. Typically, dispersion analysis is performed to study guided modes, multipolar analysis is applied to cavity modes, and both can be inspected by their near-field electromagnetic profiles [22]. Guided modes and cavity modes are both modified by a finite longitudinal thickness.

Guided mode solutions exist below the light line as continuous bands in the dispersion, meaning that they are guided in the transverse direction and generally are not accessible from light incident from the normal direction (i.e., light from the longitudinal direction) [19]. They can be classified as even or odd depending on how the phase of light is distributed throughout the structure. Likewise, they can be classified based on their incident polarization state: transverse electric (TE) or transverse magnetic (TM). When the slab is finite in the longitudinal direction, such as in a thin slab photonic crystal or dielectric metasurface, the thickness of the slab dictates the width of the bandgap. For a slab of a given periodicity, there exists an optimal thickness for which the bandgap is maximized [23].

Cavity mode solutions can exist without translational symmetry. An infinite potential well isolated from its surroundings will produce solutions independent of any neighboring structures. For the case of a sphere with a diameter on the order of the effective wavelength, the solutions for scattering follow the form of vector spherical harmonics and can be classified as transverse electric (TE) or transverse magnetic (TM), known as Mie modes [20]. However, here TE and TM Mie modes are polarization degenerate solutions that can be described as electric-type dipoles and magnetic-type dipoles, respectively. The electric and magnetic multipole solutions provide a complete basis with which to describe the radiation pattern of Mie scatterers. In the case of a dielectric cylinder illuminated with light normal to its top surface, the electric field is confined in the transverse direction for the electric Mie mode but confined in the longitudinal direction of the magnetic Mie mode (Figure 1c) [24]. The electric and magnetic Mie modes are orthogonal to one another, and the magnetic Mie mode is more sensitive to changes in the metasurface thickness due to its electric field distribution.

The description of electric-type and magnetic-type Mie modes provides an alternative perspective on the communication channels available to use in finite-thickness metasurfaces. Recently, D. Miller showed that total thickness is a limiting constraint in optics that functionally transform information, such as lenses, when considering propagation distance from input to output (or source to detector) [18]. Optics that manipulate orthogonal polarization states can double the number of communication channels available (i.e., polarization multiplexing). In photonic crystals, there are orthogonal sets of channels for TE and TM polarization states. In metasurfaces of finite thickness, there is also the opportunity to manipulate circular polarization channels due to the longitudinal degree of freedom, which is a consequence of the orthogonality of electric Mie modes from the transverse direction and magnetic Mie modes from the longitudinal direction. This describes an opportunity for metasurfaces to control twice the number of communication channels as their semi-infinite photonic crystal counterparts. We can consider the longitudinal degree of freedom as a dimension for multiplexing similar to traditional TE/TM polarization multiplexing [25].

## 3 High-Q resonances in Kerker and chiral metasurfaces

The guided modes and cavity modes of a metasurface can be coupled through careful geometric design, resulting in symmetry-protected resonances with especially long optical lifetimes (i.e., strong temporal confinement), as described by the quality factor. As discussed in the previous section, the periodicity of dielectric structures satisfies the Bloch condition and opens a bandgap of forbidden frequencies. However, small geometric perturbations can modify the band structure and generate a new set of allowed frequencies. With surprisingly small geometric perturbations, specific frequencies of light begin to constructively interfere within an otherwise forbidden bandgap, and because the existence of these resonances is dependent on the strength of the geometric perturbation, weak asymmetries result in resonances with laser-sharp linewidths and correspondingly long optical lifetimes. This phenomenon is known as symmetry-protected quasi-bound states in the continuum (q-BICs), and their emergence has been the subject of investigation in photonic crystals since at least 2008 [26–28]. The precise control of quality-factor enabled by q-BICs is especially advantageous in maximizing coupling to a material's transition energy (e.g., the linewidth of emission from a photoluminescent material, such as a quantum emitter) [29, 30].

Recently, the physics of q-BIC resonances has been combined with chiral and achiral Kerker metasurfaces to achieve high Q-factors while controlling the polarization of CPL [13, 14, 31]. The magnetic Mie mode participates in both of these phenomena. An achiral Kerker metasurface is described by orthogonal electric and magnetic Mie modes that resonate simultaneously, mimicking the requirements of the first Kerker condition (Figure 1c) [24]. A chiral metasurface is described by electric and magnetic Mie modes that are coupled as a consequence of mirror asymmetry (Figure 1d) [32]. We first describe the properties and constraints of symmetry-protected q-BIC resonances, and then detail advances in q-BIC Kerker and chiral metasurfaces.

### 3.1 High-Q resonances in metasurfaces with finite extent

The mechanism of q-BIC modes is similar to that of guided mode resonances and requires an extended optical path length [21]. The guided mode solutions become coupled to long-lived cavity mode solutions by means of a geometric perturbation. This permits otherwise dark guided modes (below the light-line) to become accessible from free space (above the light-line). Assuming infinite periodicity in the transverse direction, the quality-factor of resonances can theoretically approach infinity as the asymmetry is reduced towards zero [28]. Currently, quality factors up to 10^4^ have been experimentally demonstrated [33, 34]. Although at least one extended dimension (>100 μm at optical frequencies) is usually required to realize modes with such high quality factors, dispersion engineering techniques can be used to overcome the finite size limitations of q-BIC metasurfaces [33, 35]. So far, limitations owing to small-batch fabrication with serial lithography are the primary source of loss.

In practice, the finite extent of metasurfaces in the transverse direction imposes boundaries on the otherwise infinitely periodic guided modes. The finite metasurface area defines boundaries of a new cavity, which possesses its own cavity solutions. Recently, R. Contractor et al. have engineered the dispersion of this cavity with consideration of how to maintain single mode dispersion across the entire area [36]. They demonstrate lasing for a photonic crystal mode with effectively zero refractive index, making it immune to changes in the extent of the metasurface area. In the context of q-BIC metasurfaces, the modes originate from introducing intentional asymmetry in the in-plane (transverse) direction, and so they are also strongly affected by the unintentional asymmetry in the in-plane direction introduced by a finite area. Similar dispersion engineering techniques can be envisioned to either protect such modes from truncation (making them invariant to scaling of the metasurface area), or otherwise exploit an inhomogeneous spatial distribution for additional functionality (such as in so-called “nonlocal q-BIC metasurfaces”) [37]. Here, the infinitely periodic guided mode solutions are transformed into finite cavity mode solutions that are slowly varying and locally homogeneous only when the area is sufficiently large (>> wavelength) and geometric differences across that area are slowly varying. Relating this same concept to finite thickness, the effects of longitudinal confinement can also only be neglected in mode analysis when the thickness is sufficiently large (>> wavelength).

### 3.2 High-Q Kerker metasurfaces

**Figure 2:**
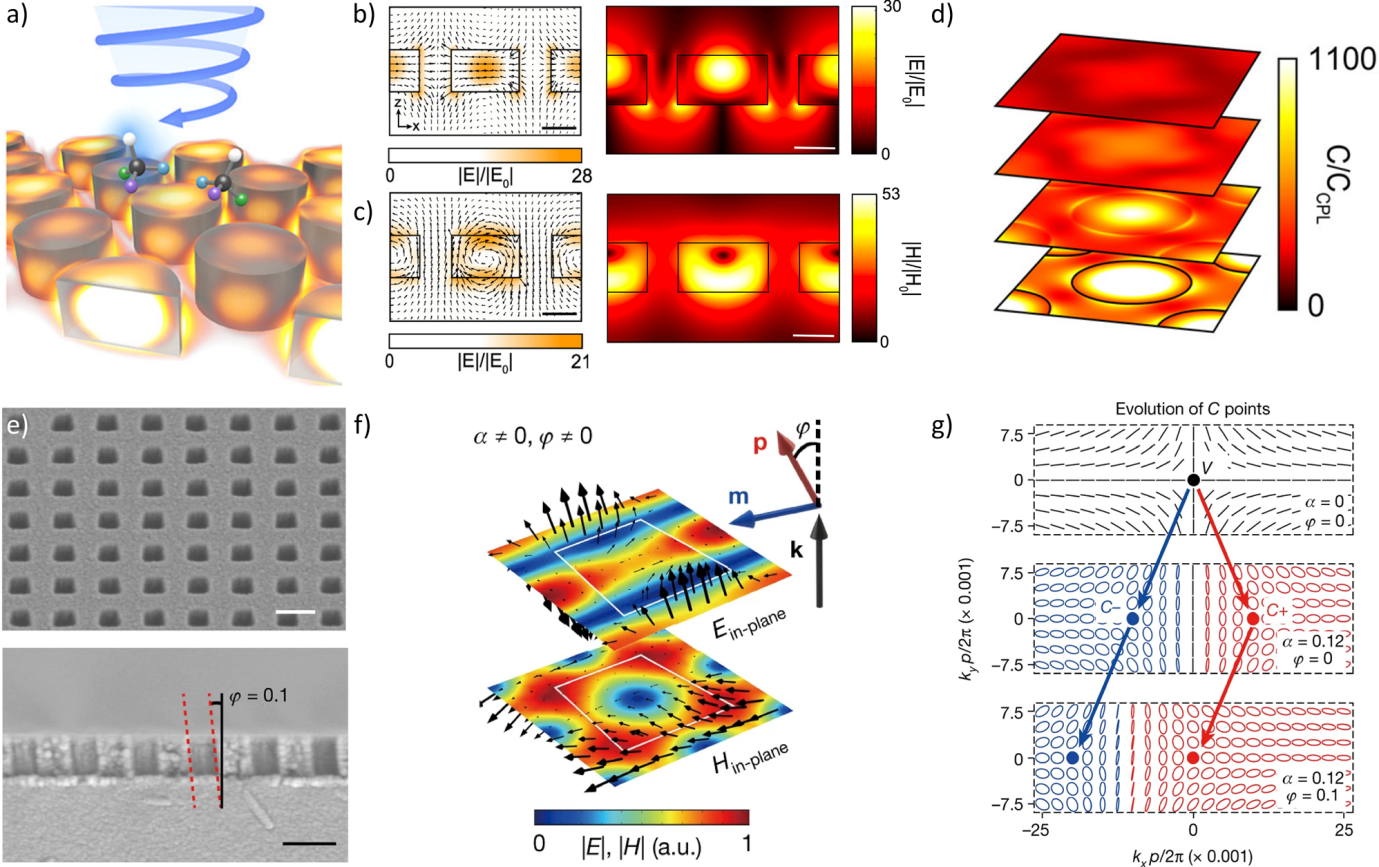
Both Kerker metasurfaces (top) and instrinsically chiral metasurfaces (bottom) rely on tuning the longitudinal dimension (thickness). (Ha–d) A high-q Kerker metasurface (Reprinted in part with permission from ACS Photonics 2020, 7, 1, 36–42 [35]): (a) schematic of decorating the Kerker metasurface with chiral biomolecules; (b) electric dipole Mie mode electric field vectors (left) and magnitude (right); (c) magnetic dipole Mie mode electric field vectors (left) and magnetic field magnitude (right); (d) enhancement in density of chirality above the metasurface when at the Kerker condition. (e–g) An intrinsically chiral high-q metasurface, where alpha is the in-plane asymmetry parameter and phi is the out-of-plane asymmetry parameter (i.e., etch slant angle) (Reprinted in part with permission from Nature 2023, 613, 474–478 [13]): (e) SEM of intrinsically chiral metasurface from top view (top) and cross section (bottom) showing a slanted etch; (f) electric (top) and magnetic (bottom) field vectors at the chiral resonance, showing coupling between the electric and magnetic fields; (g) evolution of the polarization eigenstate as a function of the etch angle (phi).

In a metasurface composed of dielectric cylinders, the spectral location of its Mie modes can be precisely tuned by the thickness (height) and diameter of the cylinders [24, 38]. In the case of increasing the diameter of the cylinder while keeping its height fixed, there is a faster spectral red-shift of the magnetic resonance frequency compared to that of the electric resonance frequency, so that modifying the height:diameter aspect ratio can be used to overlap otherwise spectrally distinct electric and magnetic Mie modes (Figure 1c). When electric and magnetic Mie modes are spectrally overlapped, interference of the resonances causes complete forward-scattering (and zero back-scattering) as a consequence of their orthogonality; such a situation is also said to satisfy the first Kerker condition, which describes the case of electric and magnetic dipoles oscillating in phase with equal magnitude [24, 39]. When Kerker metasurfaces are illuminated with circularly-polarized light, they also show enhancement of the local chiral density by several orders of magnitude while preserving the sign of the incident circularly-polarized light [40]. An analogous q-BIC metasurface is designed by breaking in-plane symmetry with a bi-periodic geometric asymmetry [41]. In this case, anti-symmetric electric and magnetic dipole resonances leak with a strength dictated by the magnitude of the asymmetry. Spectrally overlapping such high-Q resonances (again, by tuning the disk aspect ratio) leads to greater sign-preserving chiral enhancement than in the symmetric, low-Q case (Figure 2a–d) [31].

Kerker metasurface platforms can be valuable for probing chiral molecules and other light–matter interactions with chiral media [31, 40, 42, 43]. Molecular synthesis for pharmaceuticals and agrochemicals often yields enantiomers that may cause undesirable reactions in biological systems. Circular dichroism (CD) describes how chiral molecules of different handedness experience differential absorption between left and right circularly-polarized light. Techniques such as CD spectroscopy are often used to separate molecules of different handedness but require high sample concentrations due to low signals. This weak signal can be dramatically enhanced using metasurfaces that exploit the Kerker condition for maximizing the local chiral density, resulting in uniform enantiospecificity over large volumes [2, 40]. Initial experimental demonstrations have focused on enhancing fluorescence-detected circular dichroism (FDCD), which measures the difference in fluorescence intensity between right and left circularly polarized light [44]. Using a metasurface of Si nanodiscs resonant at the Kerker condition, FDCD was enhanced significantly compared to an unpatterned, nonresonant Si film which showed negligible FDCD signal [42]. Although this experiment focused on enhancing visible fluorescence, many chiral molecules are highly absorbing in the UV range. Diamond, being a high index, low loss material in the UV range, is a more suitable material platform for metasurfaces probing molecular chiral absorption. Leveraging q-BIC resonances that exhibit greater field enhancement than ordinary Mie resonances, diamond Kerker metasurfaces can enhance the local density of chirality by over three orders of magnitude (Figure 2d) [31]. This ability to control local density of chirality in a small form factor could be implemented in a pixelated design to allow multiple measurements to be made from a single, micron-scale device [35]. Additionally, these design techniques can be scaled to other wavelength ranges to enhance different spectroscopies, such as vibrational circular dichroism [45].

In addition to decorating Kerker metasurfaces with biomolecular markers, they can be interfaced with two-dimensional materials for novel schemes in data storage and transfer. For example, two-dimensional transition metal dichalcogenides (TMDCs) emit sharp and bright photoluminescence due to large exciton binding energy over 500 meV, and their broken inversion symmetry renders their electronic dispersion asymmetric at opposite *K* and *K*′ valleys, giving rise to valley-dependent circularly polarized photoluminescence [46–50]. Such valley-dependent photoluminescence can be quantified by the degree of valley polarization (DVP), defined as DVP = [*I*(*σ*+) − *I*(*σ*−)]/[*I*(*σ*+) + *I*(*σ*−)], where *I*(*σ*+/−) are right and left circularly-polarized photoluminescence intensities. The versatility of selectively addressing exciton transitions at the two valley points opens an avenue for valleytronics, spintronics, and quantum information storage. However, the short spin coherence time (a few picoseconds), due to intervalley scattering processes, results in negligible DVP at room temperature, which prevents these materials from being practically applied in opto-electronic devices. Enhancing the light–matter interactions in TMDCs is critical to obtain high DVP at high or room temperatures. Metasurfaces that strongly localize light temporally and spatially, when interfaced with TMDCs, significantly suppress the intervalley scattering processes and therefore yield strong valley-polarized emission [51, 52]. A recent demonstration of an achiral Kerker metasurface has been proposed as a new platform for enabling high DVPs for excitons (24 %) and trions (32 %) in MoS2 monolayers at 100 K [53]. The Mie modes featuring electric dipole-like and magnetic dipole-like resonances enhance the transition rates in absorption and photoluminescent emission. When both electric and magnetic dipoles are spectrally and spatially overlapped (i.e., the Kerker condition), the DVPs are maximized, paving a route towards room-temperature control of TMDC quantum emitters.

### 3.3 High-Q chiral metasurfaces

In chiral q-BIC metasurfaces intrinsically maximize circular dichroism, which in lossless (lossy) media is measured as the differential scattering (absorption) of left and right circularly polarized light. In 2D chiral metasurfaces, only mirror symmetry in the plane of the metasurface is broken. When designed into a q-BIC metasurface, inversion symmetry is also broken, and the resulting structure lacks rotational symmetry such that *C*_n_ < 3 [54]. A consequence of the reduced rotational symmetry is polarization conversion in transmission (i.e., L(R)-CP transmits to R(L)-CP), and when the metasurfaces are embedded in an index-symmetric environment, the integrated chirality over the entire volume equals zero. Planar (2D) chiral metasurfaces are not fully chiral because they still possess mirror symmetry in the out-of-plane direction, although this does not exclude their utility in controlling spin for specific applications.

Intrinsic chirality in three dimensions has recently been explored, relying on breaking both in-plane and out-of-plane mirror symmetries in the metasurface. In theory, proposed structures rely on a preserved in-plane C_2_ axis of rotational symmetry, ensuring that the metasurface behaves identically in the forward and backward directions [32, 55, 56]. In practice, 3D chirality has been achieved by etching the slab along the longitudinal direction at a precise angle (Figure 2e) and does not preserve the in-plane C_2_ axis [13, 14]. Still, optimized structures with precise tuning of the geometry (including its thickness) can minimize polarization conversion and result in near-unity circular dichroism. Q-factors nearing 10^3^ have recently been achieved experimentally in metasurfaces etched at a slanted angle [13]. Tuning the slant angle shifts one circularly polarized handedness to the Г point while pushing the other handed polarization state away from Г point, like the angular dependence of so-called extrinsically chiral metasurfaces (Figure 2f and g). These metasurfaces exhibit a nonzero integrated chirality over the volume, and intrinsically chiral photoemission has been demonstrated, opening avenues in chiral nonlinear optics. For example, combining the geometric asymmetry of a chiral metasurface with the broken time-reversal symmetry of chiral nonlinear bias imposes complete optical isolation, resulting in a singularity of the scattering matrix [32].

There is an accelerating trend in exploiting asymmetry of the longitudinal direction, all geared towards manipulating states of circularly-polarized light. These achievements may indicate a pivotal point in the design of optical cavities, where advancements in nanofabrication and materials synthesis have combined to allow for precise engineering of every dimension of a material. While most of these examples rely on a single metasurface layer, these surfaces can be stacked for similar control of the longitudinal direction [57, 58]. In comparison with the high-Q chiral metasurfaces discussed above, layered devices may lack the resolution in the longitudinal direction required for symmetry-protected high-Q resonances, and they are often made of more amorphous materials that have greater loss. Fortunately, fabrication advancements continue to make more complex and precise metamaterials more attainable; layered metasurfaces have already been demonstrated in low-Q and plasmonic designs [8–11, 59, 60].

### 3.4 Interfacing metasurfaces with quantum emitters

Metasurfaces have already proven to be a promising platform for quantum photonics, enabling manipulation and enhanced collection of the non-classical light [16, 61, 62]. In particular, photoluminescence enhancement and enhanced directional emission, tunable exciton-plasmon coupling, routing exciton emission, and separating valley excitons have been all achieved in TMDCs interfaced by metasurfaces and photonic crystals [63–67]. Integration of metasurfaces with single photon emitters as a platform for modulating and tailoring the emission will pave the way for on-chip quantum emitters en-route to designing efficient quantum metadevices. Such integration presents new opportunities in on-chip photonics and quantum information processing including quantum computation and enhanced sensing and imaging.

One direction that may be enabled by chiral or otherwise circularly birefringent metasurfaces is to achieve “spin-locking” of a quantum emitter. In general, quantum emitters can be excited by either left- or right-circularly-polarized light, and a fully symmetric optical cavity strengthens coupling of either handedness. A metasurface cavity that is selectively resonant with one handedness coupled to a quantum emitter could “lock” emission to only that handedness [68, 69]. Combined with the flexibility of metasurface design, specific areas of a free-space optical coupler could filter spin signals selectively, or store spin information selectively. Alternatively, the information could be directed to different regions of a detector in free-space. Additionally, pixelated metasurfaces would enable hyperspectral operation that compensates for the narrow bandwidth operation of high-Q resonators, drawing on work in achromatic metasurfaces [35, 70–74]. Analog optical computing architectures may be applied in quantum computing, and recent advances in these two fields simultaneously may yield multiplicative advancements towards optical quantum computers.

When integrating single photon emitters with metasurfaces, the selection rules of their coupling depend on the symmetry of their environment. Introducing intentional asymmetry can be a powerful tuning knob [75, 76]. For instance, the broken out-of-plane mirror symmetry in 2D heterolayers (e.g., Janus transition metal dichalcogenides, and Moire superlattices) has resulted in extended exciton coherence times in the nanosecond regime [77, 78]. Conversely, unintentional asymmetry can also degrade emission from quantum materials, such as through coupling with the substrate [79, 80]. As we continue to develop optical cavities for spin and chirality, both the symmetry of the cavity and the symmetry of the interacting matter should be considered simultaneously. When considered carefully, the longitudinal degree of freedom is a tool for controlling emission properties. 

## 4 Conclusions and Outlook

Quantum materials can be described by strongly correlated interactions of their lattice, charge, spin, and atomic orbitals [81]. Somewhat analogously, “quantum metasurfaces” may be described by their lattice, refractive index, spin angular momentum, and orbital angular momentum [16, 82]. An emerging platform in quantum metasurfaces may come from metasurfaces designed with their own strongly correlated properties, such as between their spin and orbital angular momentum. Just as the origin of photonic crystals was inspired by mimicking the nature of naturally occurring crystals, the next generation of quantum metamaterials may seek to mimic more recent developments in the symmetry of synthesized 2D crystals and their “twistronics”. In addition, there is a plethora of opportunity in fabricating metasurfaces directly from these new materials, providing a pathway for coupling intrinsic electronic dispersion with a nanophotonic engineered environment.

In this perspective, we have shown that the longitudinal dimension, characterized by a metasurface’s thickness, is a critical parameter in determining its interaction with circularly-polarized light. Beyond quantum optics, dielectric metasurfaces have roles in many other chiral- and spin-dependent light–matter interactions. Lorentz reciprocity, a fundamental constraint in electronics and photonics, is directly related to chiral symmetry and can be manipulated in nonlinear or time-varying metasurfaces [32, 83–86]. In communications, the dispersion of metasurfaces can be carefully engineered to design infinitely scalable lasers, or light sources that are impervious to defects (topologically protected) [36, 87, 88]. Additionally, the spin-dependent fingerprint of molecules can be enhanced for detection at low concentrations in mixtures of otherwise similar molecules, applicable across multiple spectroscopy techniques across physical chemistry and biomedicine [89–93]. The longitudinal thickness presents a degree of freedom and a dimension of symmetry often overlooked. Every dimension of an optical cavity matters, but we cannot forget its thickness.

## References

[1] Berry M. V. (2009). Optical currents. J. Opt. A: Pure Appl. Opt..

[2] Tang Y., Cohen A. E. (2010). Optical chirality and its interaction with matter. Phys. Rev. Lett..

[3] Poulikakos L., Dionne J., García-Etxarri A. (2019). Optical helicity and optical chirality in free space and in the presence of matter. Symmetry.

[4] Bliokh K. Y., Nori F. (2011). Characterizing optical chirality. *Phys. Rev. A*.

[5] Yablonovitch E. (1987). Inhibited spontaneous emission in solid-state physics and electronics. Phys. Rev. Lett..

[6] Joannopoulos J. D., Villeneuve P. R., Fan S. (1997). Photonic crystals: putting a new twist on light. Nature.

[7] Schuller J. A., Barnard E. S., Cai W., Jun Y. C., White J. S., Brongersma M. L. (2010). Plasmonics for extreme light concentration and manipulation. Nat. Mater..

[8] Zhao Y., Belkin M. A., Alù A. (2012). Twisted optical metamaterials for planarized ultrathin broadband circular polarizers. Nat. Commun..

[9] Kim R. M., Huh J. H., Yoo S. (2022). Enantioselective sensing by collective circular dichroism. Nature.

[10] Zhou S., Li J., Lu J. (2022). Chiral assemblies of pinwheel superlattices on substrates. Nature.

[11] Ma W., Cheng F., Liu Y. (2018). Deep-learning-enabled on-demand design of chiral metamaterials. ACS Nano.

[12] Kuznetsov A. I., Miroshnichenko A. E., Brongersma M. L., Kivshar Y. S., Luk’yanchuk B. (2016). Optically resonant dielectric nanostructures. Science.

[13] Chen Y., Deng H., Sha X. (2023). Observation of intrinsic chiral bound states in the continuum. Nature.

[14] Zhang X., Liu Y., Han J., Kivshar Y., Song Q. (2022). Chiral emission from resonant metasurfaces. Science.

[15] Mak K. F., Xiao D., Shan J. (2018). Light–valley interactions in 2D semiconductors. Nat. Photonics.

[16] Solntsev A. S., Agarwal G. S., Kivshar Y. S. (2021). Metasurfaces for quantum photonics. Nat. Photonics.

[17] Solomon M. L., Saleh A. A. E., Poulikakos L. V., Abendroth J. M., Tadesse L. F., Dionne J. A. (2020). Nanophotonic platforms for chiral sensing and separation. Acc. Chem. Res..

[18] Miller D. A. B. (2023). Why optics needs thickness. Science.

[19] Joannopoulos J. D., Johnson S. G., Winn J. N., Meade R. D. (2011). *Photonic Crystals*.

[20] Bohren C. F., Huffman D. R. (2008). Absorption and Scattering of Light by Small Particles.

[21] Overvig A. C., Shrestha S., Yu N. (2018). Dimerized high contrast gratings. Nanophotonics.

[22] Chai R., Liu Q., Liu W. (2023). Emerging planar nanostructures involving both local and nonlocal modes. ACS Photonics.

[23] Johnson S. G., Fan S., Villeneuve P. R., Joannopoulos J. D., Kolodziejski L. A. (1999). Guided modes in photonic crystal slabs. Phys. Rev. B Condens. Matter.

[24] Staude I., Miroshnichenko A. E., Decker M. (2013). Tailoring directional scattering through magnetic and electric resonances in subwavelength silicon nanodisks. ACS Nano.

[25] Qin H., Su Z., Liu M. (2023). Arbitrarily polarized bound states in the continuum with twisted photonic crystal slabs. Light: Sci. Appl..

[26] Marinica D. C., Borisov A. G., Shabanov S. V. (2008). Bound States in the continuum in photonics. Phys. Rev. Lett..

[27] Fedotov V. A., Rose M., Prosvirnin S. L., Papasimakis N., Zheludev N. I. (2007). Sharp trapped-mode resonances in planar metamaterials with a broken structural symmetry. Phys. Rev. Lett..

[28] Koshelev K., Lepeshov S., Liu M., Bogdanov A., Kivshar Y. (2018). Asymmetric metasurfaces with high- Q resonances governed by bound states in the continuum. *Phys. Rev. Lett.*.

[29] Thongrattanasiri S., Koppens F. H. L., García de Abajo F. J. (2012). Complete optical absorption in periodically patterned graphene. Phys. Rev. Lett..

[30] Weber T., Kuhner L., Sortino L. (2022). Strong light-matter interaction with self-hybridized bound states in the continuum in monolithic van der Waals metasurfaces. ..

[31] Hu J., Lawrence M., Dionne J. A. (2020). High quality factor dielectric metasurfaces for ultraviolet circular dichroism spectroscopy. ACS Photonics.

[32] Dixon J., Lawrence M., Barton D. R., Dionne J. (2021). Self-isolated Raman lasing with a chiral dielectric metasurface. Phys. Rev. Lett..

[33] Liu Z., Xu Y., Lin Y. (2019). High-Q quasibound states in the continuum for nonlinear metasurfaces. Phys. Rev. Lett..

[34] Lawrence M., Barton D. R., Dixon J. (2020). High quality factor phase gradient metasurfaces. Nat. Nanotechnol..

[35] Hu J., Safir F., Chang K. (2021). Rapid genetic screening with high quality factor metasurfaces. https://doi.org/10.48550/arXiv.2110.07862.

[36] Contractor R., Noh W., Redjem W. (2022). Scalable single-mode surface-emitting laser via open-Dirac singularities. Nature.

[37] So S., Mun J., Park J., Rho J. (2022). Revisiting the design strategies for metasurfaces: fundamental physics, optimization, and beyond. *Adv. Mater.*.

[38] van de Groep J., Polman A. (2013). Designing dielectric resonators on substrates: combining magnetic and electric resonances. Opt. Express.

[39] García-Etxarri A., Dionne J. A. (2013). Surface-enhanced circular dichroism spectroscopy mediated by nonchiral nanoantennas. *Phys. Rev. B*.

[40] Solomon M. L., Hu J., Lawrence M., García-Etxarri A., Dionne J. A. (2019). Enantiospecific optical enhancement of chiral sensing and separation with dielectric metasurfaces. ACS Photonics.

[41] Lawrence M., Dionne J. A. (2019). Nanoscale nonreciprocity via photon-spin-polarized stimulated Raman scattering. Nat. Commun..

[42] Solomon M. L., Abendroth J. M., Poulikakos L. V., Hu J., Dionne J. A. (2020). Fluorescence-detected circular dichroism of a chiral molecular monolayer with dielectric metasurfaces. J. Am. Chem. Soc..

[43] Ho C. S., Garcia-Etxarri A., Zhao Y., Dionne J. (2017). Enhancing enantioselective absorption using dielectric nanospheres. ACS Photonics.

[44] Tang Y., Cohen A. E. (2011). Enhanced enantioselectivity in excitation of chiral molecules by superchiral light. Science.

[45] Xiao T.-H., Cheng Z., Luo Z. (2021). All-dielectric chiral-field-enhanced Raman optical activity. Nat. Commun..

[46] Mak K. F., Lee C., Hone J., Shan J., Heinz T. F. (2010). Atomically thin MoS_2_: a new direct-gap semiconductor. Phys. Rev. Lett..

[47] Splendiani A., Sun L., Zhang Y. (2010). Emerging photoluminescence in monolayer MoS2. Nano Lett..

[48] Xiao D., Liu G.-B., Feng W., Xu X., Yao W. (2012). Coupled spin and valley physics in monolayers of MoS2 and other group-VI dichalcogenides. Phys. Rev. Lett..

[49] Mak K. F., He K., Shan J., Heinz T. F. (2012). Control of valley polarization in monolayer MoS2 by optical helicity. Nat. Nanotechnol..

[50] Cao T., Wang G., Han W. (2012). Valley-selective circular dichroism of monolayer molybdenum disulphide. Nat. Commun..

[51] Huang L., Krasnok A., Alú A., Yu Y., Neshev D., Miroshnichenko A. E. (2022). Enhanced light-matter interaction in two-dimensional transition metal dichalcogenides. Rep. Prog. Phys..

[52] Bucher T., Vaskin A., Mupparapu R. (2019). Tailoring photoluminescence from MoS_2_ monolayers by Mie-resonant metasurfaces. ACS Photonics.

[53] Liu Y., Lau S. C., Cheng W. S. (2022). Achiral dielectric metasurfaces for spectral and polarization control of valley specific light emission from monolayer MoS2. ..

[54] Shi T., Deng Z. L., Geng G. (2022). Planar chiral metasurfaces with maximal and tunable chiroptical response driven by bound states in the continuum. Nat. Commun..

[55] Overvig A., Yu N., Alù A. (2021). Chiral quasi-bound states in the continuum. Phys. Rev. Lett..

[56] Gorkunov M. V., Antonov A. A., Kivshar Y. S. (2020). Metasurfaces with maximum chirality empowered by bound states in the continuum. *Phys. Rev. Lett.*.

[57] Feis J., Beutel D., Köpfler J. (2020). Helicity-preserving optical cavity modes for enhanced sensing of chiral molecules. Phys. Rev. Lett..

[58] Zhou Y., Kravchenko I. I., Wang H., Zheng H., Gu G., Valentine J. (2019). Multifunctional metaoptics based on bilayer metasurfaces. Light: Sci. Appl..

[59] Zheng H., Zhou Y., Ugwu C. F., Du A., Kravchenko I. I., Valentine J. G. (2021). Large-scale metasurfaces based on grayscale nanosphere lithography. ACS Photonics.

[60] Tanaka K., Arslan D., Fasold S. (2020). Chiral Bilayer All-Dielectric Metasurfaces. *ACS Nano*.

[61] Qiu C.-W., Zhang T., Hu G., Kivshar Y. (2021). Quo vadis, metasurfaces?. Nano Lett..

[62] Kort-Kamp W. J. M., Azad A. K., Dalvit D. A. R. (2021). Space-time quantum metasurfaces. Phys. Rev. Lett..

[63] Lee B., Park J., Han G. H. (2015). Fano resonance and spectrally modified photoluminescence enhancement in monolayer MoS2 integrated with plasmonic nanoantenna array. Nano Lett..

[64] Li H., Wang J., Ma Y. (2020). Enhanced directional emission of monolayer tungsten disulfide (WS2) with robust linear polarization via one-dimensional photonic crystal (PhC) slab. Nanophotonics.

[65] Dibos A. M., Zhou Y., Jauregui L. A. (2019). Electrically tunable exciton-plasmon coupling in a WSe2 monolayer embedded in a plasmonic crystal cavity. Nano Lett..

[66] Wang J., Li H., Ma Y. (2020). Routing valley exciton emission of a WS2 monolayer via delocalized Bloch modes of in-plane inversion-symmetry-broken photonic crystal slabs. Light: Sci. Appl..

[67] Sun L., Wang C. Y., Krasnok A. (2019). Separation of valley excitons in a MoS2 monolayer using a subwavelength asymmetric groove array. Nat. Photonics.

[68] Li Z., Liu C., Rong X. (2018). Tailoring MoS2 valley-polarized photoluminescence with super chiral near-field. Adv. Mater..

[69] Lin W.-H., Wu P. C., Akbari H., Rossman G. R., Yeh N.-C., Atwater H. A. (2022). Electrically tunable and dramatically enhanced valley-polarized emission of monolayer WS2 at room temperature with plasmonic Archimedes spiral nanostructures. Adv. Mater..

[70] Chen W. T., Zhu A. Y., Sanjeev V. (2018). A broadband achromatic metalens for focusing and imaging in the visible. Nat. Nanotechnol..

[71] Li Z., Lin P., Huang Y. W. (2021). Meta-optics achieves RGB-achromatic focusing for virtual reality. Sci. Adv..

[72] Aieta F., Kats M. A., Genevet P., Capasso F. (2015). Applied optics. Multiwavelength achromatic metasurfaces by dispersive phase compensation. Science.

[73] Zhou Y., Kravchenko I. I., Wang H., Nolen J. R., Gu G., Valentine J. (2018). Multilayer noninteracting dielectric metasurfaces for multiwavelength metaoptics. Nano Lett..

[74] Zhu A. Y., Kuznetsov A. I., Luk’yanchuk B., Engheta N., Genevet P. (2017). Traditional and emerging materials for optical metasurfaces. Nanophotonics.

[75] Turunen M., Brotons-Gisbert M., Dai Y. (2022). Quantum photonics with layered 2D materials. Nat. Rev. Phys..

[76] Bihlmayer G., Noël P., Vyalikh D. V., Chulkov E. V., Manchon A. (2022). Rashba-like physics in condensed matter. Nat. Rev. Phys..

[77] Novoselov K. S., Mishchenko A., Carvalho A., Castro Neto A. H. (2016). 2D materials and van der Waals heterostructures. Science.

[78] Karni O., Barré E., Pareek V. (2022). Structure of the moiré exciton captured by imaging its electron and hole. Nature.

[79] Scuri G., Zhou Y., High A. A. (2018). Large excitonic reflectivity of Monolayer MoSe2 Encapsulated in hexagonal boron nitride. *Phys. Rev. Lett.*.

[80] Hayee F., Yu L., Zhang J. L. (2020). Revealing multiple classes of stable quantum emitters in hexagonal boron nitride with correlated optical and electron microscopy. Nat. Mater..

[81] Moradifar P., Liu Y., Shi J. (2022). Accelerating quantum materials development with advances in transmission electron microscopy. ..

[82] Wang K., Chekhova M., Kivshar Y. (2022). Metasurfaces for quantum technologies. Phys. Today.

[83] Koshelev K., Tang Y., Hu Z., Kravchenko I. I., Li G., Kivshar Y. (2023). Resonant chiral effects in nonlinear dielectric metasurfaces. ACS Photonics.

[84] Asadchy V. S., Mirmoosa M. S., Diaz-Rubio A., Fan S., Tretyakov S. A. (2020). Tutorial on electromagnetic nonreciprocity and its origins. Proc. IEEE Inst. Electr. Electron. Eng..

[85] Guddala S., Kawaguchi Y., Komissarenko F. (2021). All-optical nonreciprocity due to valley polarization pumping in transition metal dichalcogenides. Nat. Commun..

[86] Nefedkin N., Cotrufo M., Alù A. (2023). Nonreciprocal total cross section of quantum metasurfaces. Nanophotonics.

[87] Lu L., Joannopoulos J. D., Soljačić M. (2016). Topological states in photonic systems. Nat. Phys..

[88] Bahari B., Ndao A., Vallini F., El Amili A., Fainman Y., Kanté B. (2017). Nonreciprocal lasing in topological cavities of arbitrary geometries. Science.

[89] Dolado I., Maciel-Escudero C., Nikulina E. (2022). Remote near-field spectroscopy of vibrational strong coupling between organic molecules and phononic nanoresonators. Nat. Commun..

[90] John-Herpin A., Tittl A., Kühner L. (2022). Metasurface-enhanced infrared spectroscopy: an abundance of materials and functionalities. *Adv. Mater.*.

[91] Altug H., Oh S.-H., Maier S. A., Homola J. (2022). Advances and applications of nanophotonic biosensors. Nat. Nanotechnol..

[92] Leitis A., Tseng M. L., John-Herpin A., Kivshar Y. S., Altug H. (2021). Wafer-scale functional metasurfaces for mid-infrared photonics and biosensing. Adv. Mater..

[93] Oh S.-H., Altug H., Jin X. (2021). Nanophotonic biosensors harnessing van der Waals materials. Nat. Commun..

